# Effects of home care and e-mobile training/consultancy on women’s postpartum symptoms and breastfeeding self-efficacy: a randomized clinical trial

**DOI:** 10.1093/eurpub/ckae119

**Published:** 2024-09-09

**Authors:** Seda Karaçay Yıkar, Evşen Nazik

**Affiliations:** Department of Obstetrics and Gynecologic Nursing, Faculty of Health Sciences, Cukurova University, Faculty of Health Sciences, Adana, Sarıçam 01330, Türkiye; Department of Obstetrics and Gynecologic Nursing, Faculty of Health Sciences, Cukurova University, Faculty of Health Sciences, Adana, Sarıçam 01330, Türkiye

## Abstract

**Background:**

Most women experience breastfeeding problems and need changes due to postpartum physical symptoms and low breastfeeding self-efficacy.

**Methods:**

Postpartum home care and e-mobile training/consultancy has been introduced to address this issue. This study was conducted as a randomized controlled interventional study. The sample of the study consisted of 75 (home care group = 25, control group = 25, e-mobile training/consultancy group = 25) women who met the sample selection criteria, agreed to participate in the study, and were hospitalized in the postpartum ward of a City Hospital. Data were collected through the Personal Information Form, the Postpartum Physical Symptom Severity Scale and the Breastfeeding Self-Efficacy Scale (BSES).

**Results:**

In all postpartum stages, a statistically significant difference was detected between the home care, e-mobile training/consultancy and control group women in terms of the Postpartum Physical Symptoms Severity Scale and BSES total mean scores (*P* < .05). Interviews showed that the home care group had the lowest Postpartum Physical Symptom Severity Scale total mean scores and the highest BSES total mean scores (*P* < .05). Postpartum home visits and e-mobile training/consultancy should be considered a routine part of postpartum care. It is effective in reducing postpartum physical symptoms and increasing breastfeeding self-efficacy.

**Conclusıon:**

In line with the results of this study, the use of home care and e-mobile training/consultancy is recommended to decrease physical symptoms and increase breastfeeding self-efficacy.

Key points

- Postpartum e-mobile training/consultancy are often used by women, but few are designed and tested in research.
- It is known that women often experience physical symptoms in the postpartum period, their breastfeeding self-efficacy is low especially in the first 7 weeks and they need care in this period.
- In cases where home care services cannot be accessed in the postpartum period and in addition to home care, the use of mobile applications is very important in increasing breastfeeding self-efficacy and reducing physical symptoms.

## Introduction

The postpartum period is a sign of a new beginning for women and their families. This period involves important anatomic, physiological, psychological, and endocrine changes in the mother's body. These changes cause women to experience a set of physical symptoms. Studies that investigated the physical symptoms experienced by women in the postpartum period reported some common problems as backache, fatigue, pain in the incision/episiotomy area, dizziness, edema, hemorrhoid, constipation, urinary incontinence, sleep disorders, sexual problems, painful coitus, and breastfeeding problems [1–10]. Breastfeeding can be affected by many factors in the postpartum period, yet perceived breastfeeding self-efficacy is one of the most important ones. Mothers' breastfeeding self-efficacy should be enhanced because mothers with high self-efficacy demonstrate a positive approach by focusing on the solutions to the problems experienced during breastfeeding [11, 12].

Today, mobile devices such as smartphones and tablets have become an integral part of our daily lives and undoubtedly influenced health care delivery [13]. The increasing use of mobile devices in health care delivery has also increased the interest in mobile health applications [14, 15]. Mobile health applications providing ease of access at any place and time enable individuals to access health information, as well as monitor and share various health indicators [13]. In addition, reliable information and self-help strategies provided by these applications support individuals to better manage the postpartum period [16, 17].

Eliminating the physical symptoms and maintaining effective breastfeeding are among the main purposes of the care in the postpartum period. For this reason, providing home care and mobile support-based nursing interventions for mothers should be considered to manage the process in the postpartum period. Mobile applications for postpartum care have provided new opportunities for improving maternal and infant health services [13–15]. Studies have shown the benefits of e-mobile training/consultancy services provided in the postpartum period [18–21]. The literature includes studies indicating that mobile application training and nursing care given in the postpartum period decrease physical symptoms and increase breastfeeding self-efficacy [22, 23]. However, the literature was found to include no studies involving three groups to evaluate the management of physical symptoms and breastfeeding self-efficacy in the postpartum period.

## Methods

### Objective

This study aims to determine the effects of home care and e-mobile training/consultancy given in the postpartum period on the postpartum physical symptoms and breastfeeding self-efficacy.

### Study design and setting

This randomized parallel-group controlled trial was conducted in the postpartum service of a hospital in Adana/Turkey between June 2020 and July 2021. The study followed the Consolidated Standards of Reporting Trials (CONSORT) guidelines (CONSORT Diagram 2010) [24].

### Population and sample

The population of the study consisted of women who were vaginal delivery and hospitalized in the postpartum ward of a city hospital in the Mediterranean Region. There is no study in the literature that investigates similar variables to be taken as reference in calculating the sample size. Therefore, a pilot study was conducted. The pilot study included 30 women who gave birth vaginally (home care group: 10, control group: 10, e-mobile training/consultancy group: 10). The number of samples for the research was calculated using the G*Power 3.0.10 program. ANOVA Repeated Measures, within-between interaction (*F*) test was used for three-group analysis, and the required sample size was taken as the required sample size with 90% power, 5% margin of error and *f* = 0.148 effect size obtained as a result of the pilot study. Women recruited in the pilot study were not included in the study. The number was calculated as 63 in total, 21 in each group (*n*_1_:21; *n*_2_:21; *n*_3_:21). Considering possible data losses, the study was planned to be conducted with 87 women who met the sampling criteria (29 home care, 29 control and 29 e-mobile training/consultancy groups). The study was completed with 75 women, including 25 home care, 25 control and 25 e-mobile e-mobile training/consultancy groups (Flow Chart).

### Inclusion criteria

Women who were primigravida, gave birth vaginally, did not have multiple pregnancies, did not have a diagnosis of high-risk pregnancy, could understand and speak Turkish, and had could use smartphones (for participants in the e-mobile training/consultancy group) were included in the study.

### Exclusion criteria

Pregnant women who attended childbirth preparation classes, had known medical conditions, or had communication and psychiatric health problems were excluded. Medical conditions and psychiatric health problems were determined based on pregnant women's self-reports and medical history.

### Randomization

Block randomization was performed after the women meeting the sampling criteria were met and given information about the purpose of the study. Block randomization was performed by randomly assigning the six combinations created 15 times. The numbers 1, 2, and 3 for the home care group, control group, and e-mobile training/consultancy were assigned to the columns randomly. Which number would represent the home care group, control group, and e-mobile training/consultancy group was determined by drawing a lot at the beginning of the study. The women who were matched with the number 1 according to the draw were assigned to the home care group, those who were matched with the number 2 were assigned to the control group, and those who were matched with the number 3 were assigned to e-mobile training/consultancy group [25]. Blinding could not be implemented due to the nature of the study. However, the statistician was blinded to the evaluation of the findings.

### Data collection tools

Data were collected through the Personal Information Form, the Postpartum Symptom Severity Scale, and the Breastfeeding Self-efficacy Scale (BSES).

### Personal information form

The Personal Information Form developed by the researcher based on the related literature was composed of nine questions that collected data about women's age, education level, working condition, income level, etc [1,4,5,8,9].

### Postpartum physical symptom severity scale

The 18-item scale was developed by Chien *et al*. [26] in 2009. The scale is rated on a 4-point Likert scale, which includes options as 0 (none), 1 (mild severity), 2 (moderate severity), and 3 (high severity). The total scale score ranges between 0 and 54. Higher scores indicate higher postpartum physical symptom severity. Turkish reliability and validity of the scale were performed by Arkan and Egelioğlu Çetişli, and Cronbach's alpha value was found 0.79 [1]. This study found Cronbach's alpha value as 0.82.

### Breastfeeding Self-Efficacy Scale

The first version of the BSES developed by Dennis and Faux in 1999 includes 33 items 11. Then the 14-item short version of the form was developed in 2003. This form was responded on a 5-point Likert scale with options from 1 = “not at all confident” to 5 = “always confident”. Scores to be obtained from the scale range between 14 and 70, with higher scores indicating higher breastfeeding self-efficacy. Turkish reliability and validity of the scale were performed by Aluş Tokat, Okumuş and Dennis, and Cronbach's alpha value was found 0.86 [27]. This study found Cronbach's alpha value as 0.83.

### Data collection

The study was conducted with three groups including home care, control (standard care), and e-mobile training/consultancy groups. Data were collected via face-to-face from the women in the home care group, via the mobile application from the women in the mobile application training group, and via telephone from the women in the control group.

Each woman in the study was interviewed nine times, which included the 1st day and 3rd day and the 1st week, 2nd week, 3rd week, 4th week, 5th week, 6th week, and 7th week in the postpartum period.

### Preparation phase mobile training/consultancy application

A training booklet titled “Postpartum Period Training Booklet” was prepared considering the steps of postpartum follow-up in the Guidelines for Postnatal Care Management in Turkey (2018) [1–18, 22–28]. Written training materials were prepared in line with the literature knowledge, which included information regarding physical symptoms that can be experienced in the postpartum period, other problems in the postpartum period, and breastfeeding. The booklet was submitted to three expert lecturers to test its reliability and quality (System Usability Scale, Quality Criteria for the Health Information User (DISCERN) and Assessing the Appropriateness of Written Educational Materials Form) [29–31]. The experts found the purpose of the education booklet clear and comprehensible and the information provided in the booklet reliable and high quality. The education booklet was published after expert views were received. The postpartum period mobile application developed by the researchers is based on the Android operating system, and it can be downloaded from the Google Play Store free of charge. The application provides training about the physical symptoms experienced in the postpartum period and breastfeeding. In addition to these, the application also includes a live consultation that enables patients to communicate with researchers and an interface with questionnaires and answers to frequently asked questions by mothers.

### E-mobile training/consultancy group

On the first day of the postpartum period, the women were initially met in the hospital and informed about the purpose of the study; their written consent was received, and they were administered the Personal Information Form face-to-face. The mobile application was downloaded to the participants' devices, and the researcher provided the women with user names and passwords so that they could use the application. After the registration process on the 1st day, the participants were asked to fill in the data collection forms (Postpartum Physical Symptom Severity Scale and BSES) using the questionnaire menu in the application. The women were told that the application included information and care that they could need in the postpartum period. They were also told that they could communicate with the researcher through the mobile application anytime they needed (live support). The women in this group were contacted through the live support section in the mobile application when needed, but no phone calls or home visits were made. Women were asked to complete the Postpartum Physical Symptom Severity Scale and BSES on the 3rd day of the postpartum period and at the 1st, 2nd, 3rd, 4th, 5th, 6th, and 7th weeks in the postpartum period.

### Home care group

On the first day of the postpartum period, women were informed about the purpose of the study, their written informed consent was obtained and the Personal Information Form was applied face-to-face. Women were given care for the problems they experienced after delivery and the Postpartum Physical Symptom Severity Scale and BSES were administered. Contact and home addresses were obtained so that home care could be provided in the next interview. Home visits were made to the women on the 3rd postpartum day and in the 1st, 2nd, 3rd, 4th, 5th, 6^th^, and 7th weeks and home care was provided to reduce the physical symptoms they experienced and to increase their breastfeeding self-efficacy. Women were asked to complete the Postpartum Physical Symptom Severity Scale and BSES on the 3rd day of the postpartum period and at the 1st, 2nd, 3rd, 4th, 5th, 6th and 7th weeks in the postpartum period.

### Control group

According to the Guidelines for Postnatal Care Management in Turkey (2018), mothers should receive care three times a day in the hospital: 0–1 h, 1–6 h and 6–24 h. After discharge, they are recommended to receive care for 2–5 days, 13–17 days and 20–42 days. Women in this group received standard care in accordance with the guidelines [28]. On the first day of the postpartum period, the participants were met at the hospital and given information about the purpose of the study; their written consent was received; and they were administered the Postpartum Physical Symptom Severity Scale and BSES face-to-face. The women were provided with standard nursing care. Standard nursing care provided to women in the postpartum period was provided by clinic nurses. Contact numbers were obtained from the mothers to conduct the next interview and other interviews were conducted by telephone. The Postpartum Physical Symptom Severity Scale and BSES were administered by telephone on the 3rd day and 1st, 2nd, 3rd, 4th, 5th, 6th, and 7th weeks in the postpartum period.

### Data analysis

The obtained data were analyzed in the statistical package program ‘Statistical Package for the Social Sciences’ for Windows 24.0. The data were evaluated with descriptive statistics. Skewness and kurtosis values were used to evaluate if the data was normally distributed. Frequency tables and descriptive statistics were used to interpret the data. Three-group ANOVA test was used for parametric measurements and Kruskal–Wallis H test was used for three-group nonparametric measurements. Significant difference for three or more groups Bonferroni correction was used for pairwise comparisons of variables.

### Ethical principles of the research

Before the study was conducted, ethics approval was obtained from the Ethics Board of the School of Medicine at Cukurova University (96-55/14.02.2020); written approval was obtained from the Health Directorate of the Province of Adana; and permissions to use the scales in the study were obtained from the authors by e-mail. In addition, verbal and written consent was obtained from the participants after they were given information about the study.

## Results

The groups were found to be independent and homogeneous in terms of all descriptive characteristics (*P* > .05) (Table 1).

**Table 1.** Findings about the participants' descriptive characteristics of the groups

Group characteristics	Home care group (*n* = 25)		Control group (*n* = 25)		E-mobile training/consultancy group (*n* = 25)		Statistical analysis possibility
	*n*	%	*n*	%	*n*	%	
**Age groups**							
≤20	6	24.0	4	16.0	6	24.0	
21–23	8	32.0	8	32.0	11	44.0	χ^2^=10.296
24–26	7	28.0	8	32.0	–	–	*P* = .113
>26	4	16.0	5	20.0	8	32.0	
**Education degree**							
Literate	3	12.0	2	8.0	3	12.0	
Primary School	11	44.0	19	76.0	8	32.0	χ^2^=11.677
Bachelor’s degrees	7	28.0	3	12.0	11	44.0	*P* = .070
Graduate and above	4	16.0	1	4.0	3	12.0	
**Working status**							
Yes	3	12.0	1	4.0	3	12.0	χ^2^ = 1.261
No	22	88.0	24	96.0	22	88.0	*P* = .532
**Spouse's education status**							
Literate	–	–	1	4.0	1	4.0	
Primary School	12	48.0	13	52.0	10	40.0	χ^2^ = 4.224
Bachelor’s degrees	6	24.0	5	20.0	10	40.0	*P* = .646
Graduate and above	7	28.0	6	24.0	4	16.0	
**Occupation**							
Officer	7	28.0	6	24.0	4	16.0	
Employee	9	36.0	8	32.0	10	40.0	χ^2^ = 1.803
Self-employment	7	28.0	8	32.0	7	28.0	*P* = .937
Not working	2	8.0	3	12.0	4	16.0	
**Family type**							
Nuclear family	15	60.0	18	72.0	19	76.0	χ^2^ = 1.630
Large family	10	40.0	7	28.0	6	24.0	*P* = .443
**Economic status**							
Income < expenditure	7	28.0	7	28.0	11	44.0	χ^2^ = 3.578
Income = expenditure	16	64.0	17	68.0	14	56.0	*P* = .466
Income > expenditure	2	8.0	1	4.0	–	–	
**Planning pregnancy**							
Yes	22	88.0	24	96.0	23	92.0	χ^2^ = 1.087
No	3	12.0	1	4.0	2	8.0	*P* = .581
**Person to assist after birth**							
Mother	9	36.0	7	28.0	8	32.0	
Mother-in-law	13	52.0	12	48.0	13	52.0	χ^2^ = 9.203
Sister-in-law	–	–	4	16.0	–	–	*P* = .325
Spouse	2	8.0	1	4.0	2	8.0	
None	1	4.0	1	4.0	2	8.0	

The groups were homogeneous and indicated no statistically significant differences in terms of the Postpartum Physical Symptom Severity Scale total mean scores on the 1st day of the postpartum period (*P* > .05). A statistically significant difference was found between the groups in terms of the Postpartum Physical Symptom Severity Scale total mean scores on the 3rd day and in the 1st, 2nd, 3rd, 4th, 5th, 6th, and 7th weeks of the postpartum period (*P* = .000). Bonferroni corrected paired comparisons results showed that the significant difference was caused by the home care group and control group and mobile application training group. Home-care and mobile training/consultancy groups demonstrated significantly lower Postpartum Physical Symptom Severity Scale total mean scores in comparison to the participants in the control group on the 3rd day and in the 1st, 2nd, 3rd, 4th, 5th, 6th, and 7th weeks in the postpartum period (Table 2).

**Table 2.** Findings about the comparison of women's Postpartum Physical Symptom Severity Scale total mean scores according to their postpartum time periods

Postpartum time periods	Home care group (*n* = 25) [1]		Control group (*n* = 25) [2]		E-mobile training/consultancy group (*n* = 25) [3]		Statistical analysis *possibility
	$\bar{X}\pm S.S.$	Median [IQR]	$\bar{X}\pm S.S.$	Median [IQR]	$\bar{X}\pm S.S.$	Median [IQR]	
1.day	12.28 ± 7.36	10.0 [12.0]	13.28 ± 8.47	11.0 [8.0]	8.76 ± 5.41	8.0 [7.0]	χ^2^ = 5.443 *P* = .066
3.day	7.04 ± 5.35	5.0 [4.0]	16.60 ± 8.82	14.0 [12.0]	7.76 ± 4.82	6.0 [5.0]	χ^2^ = 24.764 *P* = .000 [2–1.3]
1.week	5.20 ± 4.85	4.0 [3.0]	14.96 ± 9.19	12.0 [12.5]	6.96 ± 3.69	6.0 [4.0]	χ^2^ = 29.756 *P* = .000 [1–2.3][2–3]
2. week	2.88 ± 1.76	3.0 [3.0]	14.20 ± 7.95	12.0 [10.0]	6.72 ± 4.33	6.0 [6.5]	χ^2^ = 38.716 *P* = .000 [1–2.3][2–3]
3. week	2.48 ± 1.78	2.0 [3.0]	10.80 ± 6.75	9.0 [9.5]	7.16 ± 5.61	5.0 [5.5]	χ^2^ = 35.188 *P* = .000 [1–2.3][2–3]
4. week	1.68 ± 1.68	1.0 [2.5]	12.56 ± 6.25	11.0 [8.0]	7.12 ± 6.04	7.0 [8.0]	χ^2^ = 43.401 *P* = .000 [1–2.3][2–3]
5. week	0.92 ± 1.04	1.0 [1.5]	12.72 ± 5.60	11.0 [8.0]	6.44 ± 5.53	5.0 [8.5]	χ^2^ = 46.934 *P* = .000 [1–2.3][2–3]
6. week	0.72 ± 1.02	0.0 [1.5]	11.68 ± 6.27	10.0 [11.5]	5.44 ± 4.55	5.0 [8.5]	χ^2^ = 42.655 *P* = .000 [1–2.3][2–3]
7. week	0.56 ± 0.83	0.0 [1.0]	11.88 ± 6.05	10.0 [11.5]	5.12 ± 4.29	5.0 [8.5]	χ^2^ = 45.871 *P* = .000 [1–2.3][2–3]

* “Kruskal–Wallis H” test (χ2-table value) statistics were used to compare the measurement values of three or more independent groups with no normal distribution.

The groups were homogeneous and had no significant differences in terms of their BSES total mean scores on the 1st day of the postpartum period (*P* > .05). A statistically significant difference was found between the groups in terms of their BSES total mean scores in the 2nd, 3rd, 4th 5th, 6th, and 7th weeks in the postpartum period (*P* = .000). Bonferroni corrected paired comparisons results showed that the difference was between the home care group and the control group and the mobile application training group. BSES total mean scores of the home care group were found to be significantly higher than the control group and mobile training/consultancy group. In a similar vein, a significant difference was found between the e-mobile training/consultancy group and the control group. BSES total mean scores of the e-mobile training/consultancy group were found to be significantly higher in comparison to the participants in the control group (Table 3).

**Table 3.** Findings about the comparison of women's BSES total mean scores according to the postpartum time periods

Postpartum time periods	Home care group (*n* = 25) [1]		Control group (*n* = 25) [2]		E-mobile training/consultancy group (*n* = 25) [3]		Statistical analysis*possibility
	$\bar{X}\pm S.S.$	Median [IQR]	$\bar{X}\pm S.S.$	Median [IQR]	$\bar{X}\pm S.S.$	Median [IQR]	
1. Day	46.92 ± 6.51	47.0 [8.5]	45.28 ± 7.81	46.0 [11.0]	50.64 ± 8.96	50.0 [13.5]	*F* = 3.080 *P* = .052
3. Day	59.28 ± 7.37	58.0 [13.0]	46.56 ± 9.34	46.0 [13.0]	58.40 ± 7.78	59.0 [13.0]	*F* = 18.730 *P* = .000 [2–1.3]
1. Week	62.16 ± 6.57	66.0 [11.0]	49.12 ± 9.77	49.0 [12.5]	59.48 ± 9.84	62.0 [13.5]	χ^2^ = 21.744 *P* = .000 [2–1.3]
2. Week	66.04 ± 4.55	66.0 [3.0]	45.44 ± 8.85	46.0 [15.0]	60.84 ± 5.96	61.0 [10.5]	χ^2^ = 45.716 *P* = .000 [1–2.3][2–3]
3. Week	66.04 ± 4.28	66.0 [4.0]	46.12 ± 9.10	46.0 [16.5]	60.56 ± 6.44	60.0 [12.0]	χ^2^ = 43.658 *P* = .000 [1–2.3][2–3]
4. Week	66.32 ± 4.18	66.0 [4.0]	46.16 ± 8.06	46.0 [13.0]	61.80 ± 5.07	62.0 [8.5]	χ^2^ = 49.805 *P* = .000 [1–2.3][2–3]
5. Week	67.40 ± 2.58	67.0 [4.0]	45.88 ± 8.37	49.0 [14.5]	60.96 ± 7.66	63.0 [10.5]	χ^2^ = 49.984 *P* = .000 [1–2.3][2–3]
6. Week	67.68 ± 2.30	67.0 [4.0]	45.92 ± 7.10	46.0 [10.0]	61.88 ± 7.53	66.0 [8.5]	χ^2^ = 52.549 *P* = .000 [1–2.3][2–3]
7. Week	67.88 ± 1.88	67.0 [4.0]	46.08 ± 7.05	49.0 [9.5]	62.16 ± 7.66	66.0 [8.0]	χ^2^ = 52.138 *P* = .000 [1–2.3][2–3]

* “ANOVA” test (*F*-table value) statistics were used to compare the measurement values of three or more independent groups with normal distribution. “Kruskal-Wallis H” test (χ^2^-table value) statistics were used to compare the measurement values of three or more independent groups that do not have normal distribution.

Home-care and control group participants were found to have no statistically significant relationship in terms of their last interview and Postpartum Physical Symptom Severity Scale and BSES total mean scores (*P* > .05). The e-mobile training/consultancy group was found to have a negative, weak, and statistically significant relationship in terms of their last interview and Postpartum Physical Symptom Severity Scale and BSES total mean scores (*r* = −0.446; *P* = .025). BSES total mean scores would increase with the decrease in the last interview Postpartum Physical Symptom Severity Scale mean scores (Table 4).

**Table 4.** Findings about the relationship between the participants' last interview and Postpartum Physical Symptom Severity Scale and BSES total mean scores

Correlation*	Postpartum Physical Symptom Severity Scale					
	Home care group (*n* = 25)		Control group (*n* = 25)		E-mobile training/consultancy group (*n* = 25)	
	*r*	*P*	*r*	*P*	*r*	*P*
**BSES**	−0.037	.859	0.145	.489	−0.446	.025

* “Spearman” correlation coefficient was used to examine the relationships of two quantitative variables that do not have normal distribution.

## Discussion

Women experience some physical symptoms and breastfeeding problems. Chan reported that home care was effective in eliminating the postpartum period problems of the women who were provided with home care. Quality and repetitive care provided for women's needs can be considered to help women to get used to this process and experience fewer physical symptoms [32].

Women in the mobile training/consultancy group were found to have significantly lower Postpartum Physical Symptom Severity Scale mean scores in comparison to the control group. Nair *et al*., Sawyer *et al*., and Ju Yeon and Hye Young reported that the use of mobile applications in the postpartum period was effective in decreasing depression symptoms [33–35]. The study findings indicate the effects of forming adequate and equipped applications on women's health in the postpartum period. Well-designed mobile application health services designed for women's needs are reported to help women to experience fewer physical symptoms in the postpartum period and get used to this process. The use of the web, telephone, or video conference techniques to support this process enables to access mothers who have care needs in cases like pandemics when home visits cannot be made and in cases when the number of health personnel is insufficient.

Breastfeeding self-efficacy was found to be the highest in the home care group. This finding indicates that mothers' self-efficacy can be increased by evaluating them in the environments where they live and encouraging their participation in the process, regulating breastfeeding by managing the process together, and performing repetitive education and consultancy services. In a similar vein, Chan *et al*., Tatar, and Kocagöz Acar found that repetitive breastfeeding consultancy given in the postpartum period increased breastfeeding self-efficacy [36–38].

BSES total mean scores of the women in the e-mobile training/consultancy group were found to be higher in comparison to the women in the control group. This finding shows that the e-mobile training/consultancy given in the postpartum period was beneficial for breastfeeding self-efficacy. Meedya *et al*. and Lewkowitz *et al*. reported that self-efficacy increased as a result of the education given. The mobile health application, which is easily used and always accessible whenever mothers need it, seems to increase mothers' self-efficacy mean scores by increasing women's self-confidence through the consultancy provided via the application [39, 40].

BSES total mean scores of the women in the home care and mobile training/consultancy group decreased with the increase in the Postpartum Physical Symptom Severity Scale mean scores; only the e-mobile training/consultancy group was found to demonstrate a negative, weak, and statistically significant relationship (*P* < .05). This finding indicates the need for eliminating the physical symptoms experienced by women to increase their breastfeeding self-efficacy. Similarly, Egelioğlu Çetişli *et al*. evaluated the effect of postpartum physical symptoms on breastfeeding success and reported that breastfeeding success decreased with the increase in the symptoms [3]. Mothers could be affected by this process more due to the obscurity of the process and the increased responsibilities they have. Breastfeeding is a highly important issue in our society. In some parts of society, some people believe that breastfeeding women are better mothers. Mothers' breastfeeding self-efficacy decreases due to these judgments and the physical symptoms they experience. Therefore, regular home visits and the e-mobile applications developed are believed to be effective in including mothers in this process as soon as possible for the solution to this problem.

## Strengths and limitations

The interventions were performed during the peak of the COVID-19 pandemic and the women’s care was done in the postpartum period via home visits and mobile application shows the strength of the study.

There were certain limitations of this study. The first limitation is cannot be blinded due to the nature of the study performed. The second limitation is that outcomes of physical symptoms and breastfeeding experiences in the postpartum period are based on participants self-reports. This study can only be generalized to women who had a normal vaginal delivery. Women may experience physical symptoms and problems with breastfeeding within one year postpartum. Seven weeks postpartum is another limitation. It is not clear that similar results will occur in the postpartum period.

## Conclusions and recommendations

Home care and e-mobile training/consultancy services given to women in the postpartum period were found to decrease physical symptoms and increase breastfeeding self-efficacy. In line with these results, it is recommended to provide women in the postpartum period with planned trainings about the problems experienced in the postpartum period through home visits performed at the earliest time possible and provide them with consultancy services based on their needs. Using mobile applications in the postpartum period when home visits are not possible may be an alternative choice. It is recommended that integrate mobile applications developed for the postpartum period with hospital-based programs for the women's use. Plan studies that test the effect of mobile application on physical symptoms and breastfeeding self-efficacy over one year since the problems in the postpartum period could maintain up to 1 year.

## Supplementary Material

ckae119_Supplementary_Data

## Data Availability

The data underlying this article will be shared on reasonable request to the corresponding author.

## References

[1] Arkan G , Egelioğlu Cetişli N. Validity and reliability study of postpartum physical symptom severity scale. Int Peer-Rev J Gynecol Matern Child Health 2017;10:18–34.

[2] Wilkie S , Crawley R, Button S et al Assessing physical symptoms during the postpartum period: reliability and validity of the primary health questionnaire somatic symptom subscale (PHQ-15). J Psychosom Obstet Gynaecol 2018;39:56–63. 10.1080/0167482X.2017.1289167. 28635532

[3] Egelioğlu Cetişli N , Işık S, Kahveci M et al Postpartum physical symptom severity and breastfeeding behaviour of primipar mother according to their birth type. HEAD 2020;7:98–103. 10.5222/HEAD.2020.68095.

[4] Aksu S , Çatalgöl Ş. Long term postpartum health physical problems of womens and associations with self-rated health. J Women Health Nurs 2017;3:22–42.

[5] Bağcı S , Altuntuğ K. Problems experienced by mothers in postpartum period and their associations with quality of life. J Human Sci 2016;13:3266–79. 10.14687/JHS.V13I2.3884.

[6] Kohler K , Annerstedt S, Diwan V et al Postpartum quality of life in Indian women after vaginal birth and cesarean section: a pilot study using the EQ-5D-5L descriptive system. BMC Pregnancy Childbirth 2018;18:427. 10.1186/s12884-018-2038-0. 30373545 PMC6206933

[7] Fata S , Atan ŞÜ. The relationship between fatigue and breastfeeding self-efficacy. Niger J Clin Pract 2018;21:1408–14. 10.4103/njcp.njcp_366_17. 30417837

[8] Kirlek F , Akdolun-Balkaya N. The effects of breast milk and olive oil on prevention of nipple pain and nipple cracks at early postpartum period. Assoc Res Dev Nurs 2013;15:17–34.

[9] Haran C , Driel M, Mitchell BL et al Clinical guidelines for postpartum women and infants in primary care–a systematic review. BMC Pregnancy Childbirth 2014;14:51. 10.1186/1471-2393-14-51. 24475888 PMC3906750

[10] Song J , Chae HJ, Kim CH. Changes in perceived health status, physical symptoms, and sleep satisfaction of postpartum women over time. Nurs Health Sci 2014;16:335–42. 10.1111/nhs.12109. 24372842

[11] Dennis C , Faux S. Development and psychometric testing of the breastfeeding self-eﬃcacy scale. Res Nurs Health 1994;22:399–409. 10.1002/(sici)1098-240x(199910)22:5<399::aid-nur6>3.0.co;2-4. 10520192

[12] Yenal K , Aluş Tokat M, Durgun Ozan Y et al The relation between breastfeeding self-efficacy and breastfeeding success in mothers. Assoc Res Dev Nurs 2013;10:14–9.

[13] Doğan Merih Y , Karabulut Ö, Gülşen Ç. The effect of mother ınfant school on postpartum adaptation and mothers' satisfaction. Turk J Res Dev Nurs 2017;19:21–33.

[14] Grier G , Geraghty S. Mind matters: developing skills and knowledge in postnatal depression. British Journal Of Midwifery 2015;23:110–4. 10.12968/bjom.2015.23.2.110.

[15] Derbyshire E , Dancey D. Smartphone medical applications for women’s health: what ıs the evidence-base and feedback? Int J Telemed Appl 2013;2013:782074–9. 10.1155/2013/782074. 24454354 PMC3880694

[16] Palacı H , Yarar O, Kuru İ et al Evaluation of smart phone applications’ validity, certifcation, security and users adoptation perspective. Med Technol Congr 2016;308–311.

[17] Liu P , Astudillo K, Velez D et al Use of mobile health applications in low-income populations. Circ Cardiovasc Qual Outcomes 2020;13:e007031. https://doi.org/10. 1161/CIRCOUTCOMES.120.007031. 32885681 10.1161/CIRCOUTCOMES.120.007031

[18] Chamangasht M , Akbari Kamrani M, Farid M. Efficacy of an early self-care-based education program on the self-evaluation of primiparous postpartum mothers: a randomized controlled clinical trial. Shiraz E-Med J 2021;22:1–7 10.5812/semj.108132.

[19] Nasir S , Goto R, Kitamura A et al Dissemination and implementation of the e-MCHHandbook, UNRWA’s newly released maternal and child health mobile application: a cross-sectional study. BMJ Open 2020;10:e034885. 10.1136/bmjopen-2019-034885. PMC7064073 32156767

[20] Wirsiy FS , Ako-Arrey DE, Njukeng PA. Mobile health ınterventions in Cameroon: a review of their effect on women’s health outcomes. J Womens Health Dev 2019;2:076–89. 10.26502/fjwhd.2644-28840010.

[21] Mckay FH , Cheng C, Wright A et al Evaluating mobile phone applications for health behaviour change: a systematic review. J Telemed Telecare 2018;24:22–30. 10.1177/1357633X16673538. 27760883

[22] Lavender T , Richens Y, Milan SJ, Cochrane Pregnancy and Childbirth Group et al Telephone support for women during pregnancy and the first six weeks postpartum (Review). Cochrane Database Syst Rev 2013;2013:1–67 10.1002/14651858.CD009338.pub2. PMC8078598 23881662

[23] Brodribb W , Hawley G, Mitchell M et al Face-to-face health professional contact for postpartum women: a systematic review. Women Birth 2020;33:e492–e504. 10.1016/j.wombi.2019.11.003. 31859253

[24] Consort Diagram. 2010. https://www.consort-spirit.org/ (3 July 2024, date last accessed).

[25] Random List. https://www.random.org/ (3 July 2024, date last accessed).

[26] Chien LY , Tai CY, Hwang FM et al Postpartum physical symptoms and depressive symptomatology at 1 month and 1 year after delivery: a longitudinal questionnaire survey. Int J Nurs Stud 2009;46:1201–8. 10.1016/j.ijnurstu.2009.02.007. 19278680

[27] Tokat MA , Okumuş H, Dennis CL. Translation and psychometric assessment of the breast-feeding self-efficacy scale-short form among pregnant and postnatal women in Turkey. Midwifery 2010;26:101–8. 10.1016/j.midw.2008.04.002. 18541350

[28] Guidelines for Postnatal Care Management in Turkey. 2018. https://hsgm.saglik.gov.tr/depo/Yayinlarimiz/Rehberler/DOGUM_SONU_BAKIM_08-01-2019_1.pdf (3 July 2024, date last accessed).

[29] Demirkol D , Seneler ÇA. Turkish Translation Of The System Usability Scale: the SUSTR. Usak Univ J Soc Sci 2018;11:237–53. 10.29217/uujss.497.

[30] Charnock D , Shepperd S, Needham G et al DISCERN: an instrument for judging the quality of written consumer health information on treatment choices. J Epidemiol Community Health 1999;53:105–11. 10396471 10.1136/jech.53.2.105 PMC1756830

[31] Demir F , Ozsaker E, Ilce AO. Özcan-Ilce A. The quality and suitability of written educational materials for patients. J Clin Nurs 2008;17:259–65. 10.1111/j.1365-2702.2007.02044.x. 18171395

[32] Chan P. Postpartum care service experience model: a case of postpartum home visiting services. Healthcare 2018;6:91. 10.3390/healthcare6030091. 30071653 PMC6165335

[33] Nair U , Armfield NR, Chatfield MD et al The effectiveness of telemedicine interventions to address maternal depression: a systematic review and meta-analysis. J Telemed Telecare 2018;24:639–50. 10.1177/1357633X18794332. 30343660

[34] Sawyer A , Kaim A, Le HE et al Evaluating the effectiveness of an app-based nurse-moderated program for new mothers with depression and parenting problems (Emums Plus): protocol for a pragmatic randomized controlled trial. JMIR Res Protoc 2019;8:e11549. 10.2196/13689. 30664487 PMC6351991

[35] Ju Yeon L , Hye Young K. Development and validation of a postpartum care mobile application for first-time mothers. Korean J Women Health Nurs 2017;23:210–20. 10.4069/kjwhn.2017.23.3.210. 37684900

[36] Chan MY , Ip WY, Choi KC. The effect of a self-efficacy-based educational programme on maternal breast feeding self-efficacy, breast feeding duration and exclusive breast feeding rates: a longitudinal study. Midwifery 2016;36:92–8. 10.1016/j.midw.2016.03.003. 27106949

[37] Tatar B. The effect of breastfeeding education on postnatal breastfeeding nulliparous pregnant women. Ph.D. Thesis, Istanbul University Health Institute, 2019.

[38] Kocagöz Acar G. Evaluation of the effect milk counseling to mothers of preterm infants in the postpartum period on breastfeeding perception and adequacy. Ph.D. Thesis, Bulent Ecevit University Health Institute, 2021.

[39] Meedya S , Win K, Yeatman H et al Developing and testing a mobile application for breastfeeding support: the milky way application. Women Birth 2021;34:e196–e203. 10.1016/j.wombi.2020.02.006. 32081557

[40] Lewkowitz AK , Cahill AG. Mobile health approaches to breastfeeding. Clin Obstet Gynecol 2021;64:384–91. 10.1097/GRF.0000000000000606. 33813523

