# Supplementary material for: Effects of home care and e-mobile training/consultancy on women’s postpartum symptoms and breastfeeding self-efficacy: a randomized clinical trial
Source: Eur J Public Health. 2024 Sep 9;34(5):936–42. doi: 10.1093/eurpub/ckae119 (PMC11430928; doi:10.1093/eurpub/ckae119)
Supplement: ckae119_Supplementary_Data [file ckae119_supplementary_data.zip › ckae119_Supplementary_Data/ejph-2024-05-om-0313-File002.docx]

| **Creation of groups** | | |
| --- | --- | --- |
| **E‑Mobile Training/Consultancy group (n=25)** | **Home-care group** **(n=25)** | **Control group (n=25)** |
| 1.interview (in the hospital on the 1st day in the postpartum period)   - Written consetnt form - Personal information form Postpartum physical symptom severity scale - BSES | 1.interview (in the hospital on the 1st day in the postpartum period))   - Written consetnt form - Personal information form Postpartum physical symptom severity scale - BSES | 1.interview (in the hospital on the 1st day in the postpartum period))   - Written consetnt form - Personal information form Postpartum physical symptom severity scale - BSES |
| 2.interview (1st week postpartum 3rd day via mobile application)   - Postpartum physical symptom severity scale - BSES | 2.interview (Postpartum in the 1st week, at home on the 3rd day)  Postpartum physical symptom severity scale   - BSES | 2.interview (By phone on the 3rd day after birth in the 1st week)   - Postpartum physical symptom severity scale - BSES |
| 3.interview (1st week postpartum via mobile application)   - Postpartum physical symptom severity scale - BSES | 3.interview (at home in week 1)   - Postpartum physical symptom severity scale - BSES | 3.interview (1 week by phone)   - Postpartum physical symptom severity scale - BSES |
| 4.interview (2nd week via mobile app)   - Postpartum physical symptom severity scale - BSES | 4.interview (at home in week 2)   - Postpartum physical symptom severity scale - BSES | 4.interview (2 week by phone)   - Postpartum physical symptom severity scale - BSES |
| 5.interview (3nd week via mobile app)   - Postpartum physical symptom severity scale - BSES | 5.interview (at home in week 3)   - Postpartum physical symptom severity scale - BSES | 5.interview (3 week by phone)   - Postpartum physical symptom severity scale - BSES |
| 6.interview (4nd week via mobile app)   - Postpartum physical symptom severity scale - BSES | 6.interview (at home in week 4)   - Postpartum physical symptom severity scale - BSES | 6.interview (4 week by phone)   - Postpartum physical symptom severity scale - BSES |
| 7.interview (5nd week via mobile app)   - Postpartum physical symptom severity scale - BSES | 7.interview (at home in week 5)   - Postpartum physical symptom severity scale - BSES | 7.interview (5 week by phone)   - Postpartum physical symptom severity scale - BSES |
| 8.interview (6nd week via mobile app)   - Postpartum physical symptom severity scale - BSES | 8.interview (at home in week 6)   - Postpartum physical symptom severity scale - BSES | 8.interview (6 week by phone)   - Postpartum physical symptom severity scale - BSES |
| 9.interview (7nd week via mobile app)   - Postpartum physical symptom severity scale - BSES | 9.interview (at home in week 7)   - Postpartum physical symptom severity scale - BSES | 9.interview (7 week by phone)   - Postpartum physical symptom severity scale - BSES |

Flow chart
