# Supplementary material for: Effects of home care and e-mobile training/consultancy on women’s postpartum symptoms and breastfeeding self-efficacy: a randomized clinical trial
Source: Eur J Public Health. 2024 Sep 9;34(5):936–42. doi: 10.1093/eurpub/ckae119 (PMC11430928; doi:10.1093/eurpub/ckae119)
Supplement: ckae119_Supplementary_Data [file ckae119_supplementary_data.zip › ckae119_Supplementary_Data/ejph-2024-05-om-0313-File003.doc]

**Allocation**

**Analysis**

**Follow-Up**

**Enrollment**

Assessed for eligibility (n=168)

Excluded (n=81)

♦  Not meeting inclusion criteria (n=69)

♦  Declined to participate (n=12)

Analysed (n=25)
♦ Excluded from analysis (n=0)

Lost to follow-up (n=4)

Didn't continue the intervention (n=2)

Complication has developed (n=2)

E-mobile training/consultancy group (n=29)

Received allocated intervention (n=29)

♦ Did not receive allocated intervention (n=1)

Lost to follow-up (n=1)

♦ Didn't continue the intervention (n=3)

Control (standard care) group:

Received allocated intervention (n=29)

♦ Did not receive allocated intervention (n=0)

Analysed (n=25)
♦ Excluded from analysis (n=0)

Randomized (n=87)

Home care group

(n=29)

 Received allocated intervention (n=29)

♦ Did not receive allocated intervention (n=0)

Analysed (n=25)
♦ Excluded from analysis (n=0)

CONSORT Diagram 2010

Lost to follow-up (n=4)

Didn't continue the intervention (n=2)

Contact address not available (n=2)
